# Neurodegenerative Diseases in Male Former First-Class New Zealand Rugby Players

**DOI:** 10.1007/s40279-025-02299-y

**Published:** 2025-09-04

**Authors:** Francesca Anns, Kenneth L. Quarrie, Barry J. Milne, Chao Li, Andrew J. Gardner, Ian R. Murphy, Evert Verhagen, Craig Wright, Susan M. B. Morton, Thomas Lumley, Lynette Tippett, Stephanie D’Souza

**Affiliations:** 1 Centre of Methods and Policy Application in the Social Sciences, Faculty of Arts and Education, University of Auckland, Auckland, New Zealand; 2 School of Psychology, Faculty of Science, University of Auckland, Auckland, New Zealand; 3 New Zealand Rugby, Wellington, New Zealand; 4 Sports Performance Research Institute New Zealand (SPRINZ), Auckland University of Technology, Auckland, New Zealand; 5 Auckland Bioengineering Institute (ABI), The University of Auckland, Auckland, New Zealand; 6 School of Social Sciences, Faculty of Arts and Education, University of Auckland, Auckland, New Zealand; 7 Sydney School of Health Sciences, Faculty of Medicine and Health, University of Sydney, Camperdown, NSW Australia; 8 Illawarra-Shoalhaven Local Health District, New South Wales Health, Warrawong, NSW Australia; 9 Amsterdam Collaboration on Health and Safety in Sports, Department of Public and Occupational Health, Amsterdam Movement Sciences, Amsterdam UMC, Amsterdam, The Netherlands; 10 Social Investment Agency, Wellington, New Zealand; 11 Research Institute for Innovative Solutions for Well-being and Health, Faculty of Health, University of Technology, Sydney, Australia; 12 Department of Statistics, Faculty of Science, University of Auckland, Auckland, New Zealand

## Abstract

**Background:**

Growing concern surrounds the risk of neurodegenerative diseases in high-level collision sports, but research on Rugby Union's connection to these diseases is limited.

**Objective:**

This study sought to examine the long-term neurodegenerative disease risk associated with participation in high-level Rugby Union (‘rugby’), utilising whole-population administrative records.

**Methods:**

This retrospective cohort study in New Zealand compared males born between 1920 and 1984 who were active in high-level (provincial or higher) rugby between 1950 and 2000 (*n* = 12,861) with males from the general population (*n* = 2,394,300), matched by age, ethnicity, and birthplace. We used Cox proportional hazards models to assess risks of Alzheimer's disease, Parkinson's disease, motor neuron disease, and other dementias, ascertained using mortality and hospitalisation records from January 1988 to June 2023.

**Results:**

A higher percentage of rugby players (6.5%) than males in the general population (5.2%) developed neurodegenerative diseases, with hazard ratios indicating players showed increased risks for any neurodegenerative disease (1.22; 95% confidence interval [CI] 1.14–1.30), Alzheimer's disease (1.61; 95% CI 1.42–1.83), and other dementias (1.23; 95% CI 1.14–1.33). Significant differences were not observed for Parkinson’s disease (1.05; 95% CI 0.89–1.22) and motor neuron disease (1.16; 95% CI 0.83–1.63). In general, this increased risk among players compared to the general population began around the ages of 70–79 years. Compared to the general population, small to moderate increased risks of any neurodegenerative disease were observed for a backline playing position, provincial and/or amateur players, international and/or professional players, participation in ≥ 2 years of play, and participation in five or more matches.

**Conclusions:**

High-level rugby participation amongst males in New Zealand is associated with a small to moderate increase in neurodegenerative disease rates compared to the general population.

**Supplementary Information:**

The online version contains supplementary material available at 10.1007/s40279-025-02299-y.

## Key Points

**Table:** 

Former male Rugby Union players are at increased risk of neurodegenerative disease, particularly Alzheimer’s disease and other dementias, compared to males in the general population.
Greater risks were observed for individuals with moderate-to-high exposure to first-class rugby and backline players relative to general population males.
These findings may not be generalisable to female players, players at lower levels, and first-class rugby in more recent years.

## Introduction

Team collision sports involve impacts with other players or the playing surface, increasing exposure to head acceleration events (HAEs) and, potentially, brain injuries—an often-cited preventable risk factor for dementia. However, findings and evidence quality remain mixed [1–4]. Concern about the possible long-term effects of mild traumatic brain injuries (TBIs) and repeated HAEs associated with collision sports has increased over the past 2 decades following the discovery at autopsy of chronic traumatic encephalopathy neuropathologic change (CTE-NC) in the brains of former athletes [5]. It has been postulated that CTE-NC marks a progressive neurodegenerative disease unique to those with repeated brain trauma [5]. Nevertheless, there remains debate over the clinical and pathological features of CTE-NC, and understanding of the disease and its association with repeated brain trauma is limited in part due to the study designs used (primarily cross-sectional and case-series) and the large degree of selection bias in many studies to date [6–8].

Beyond chronic traumatic encephalopathy, two recent systematic reviews of retrospective cohort studies have highlighted a consistent association between exposure to contact and collision sports at the professional and/or elite level of participation and an increased risk of incidence and death from neurodegenerative diseases compared to individuals in the general population [9, 10]. For example, studies of Association Football (soccer) and American Football have found higher rates of mortality from neurodegenerative diseases among former international or professional athletes than general population comparisons [11–14].

Leading hypotheses postulate that exposure to repeated HAEs may be an important consideration in understanding the mechanisms underlying the association between contact and collision sport and neurodegenerative disease risk [6, 15–17]. Other mechanisms proposed to influence or explain the association between contact and collision sport include reactivation of herpes simplex virus type 1 (HSV-1) in the brains of *APOE4* carriers resulting from repeated brain injuries, use of painkillers and anaesthetics, use of alcohol, ergogenic aids (e.g. performance-enhancing drugs), and, for field sports, exposure to soil borne pathogens or pesticides [18–21]. Fourteen potential modifiable risk factors have recently been described by the Lancet Commission on dementia, one of which is traumatic brain injury [22].

Rugby Union (‘rugby’) is a popular collision sport with one of the highest rates of mild TBI [2, 23, 24].

While participation in sport and other forms of physical activity has been associated with significant health benefits [25, 26], long-term outcomes associated with participation in collision sports such as rugby may include neurocognitive deficits [27–32]. A 2018 review of the relationship between playing rugby and later-life brain health reported that there is some evidence of significantly decreased performance on measures of fine motor control and increased self-reported cognitive complaints among retired rugby players compared to control populations, although the literature remains mixed overall [33]. Additionally, neuroimaging studies have shown evidence of potential abnormalities in the brain structure of rugby players [34–36]. However, most studies that have investigated the neurodegenerative disease risk associated with participation in rugby are cross-sectional and many have used opt-in designs, which may increase the likelihood of selection bias. A notable exception is a 2022 retrospective cohort study by Russell et al., which found a 2.67 (95% confidence interval [CI] 1.67–4.27) times higher rate of incident neurodegenerative diseases among 412 former international rugby players from Scotland compared to a population comparison group (*n* = 1236) [37]. Therefore, longitudinal research on the health outcomes associated with participation in high-level rugby remains limited.

Evidence is also mixed regarding the risk of neurodegenerative diseases associated with sport-specific factors linked to greater repetitive HAEs [9, 10]. These factors include position played, level of play (professional versus amateur), and career duration. For example, in rugby, players in a forward position are generally exposed to more contact events per match than backline players [38], and recent studies using instrumented mouthguards to monitor head accelerations in elite rugby players have reported that forwards have higher rates of head accelerations per match than backs (22.7 per hour for forwards, 13.2 per hour for backs) [39]. However, players in backline positions may experience a greater risk of injury (including head injuries) when exposed to contact events [38–42]. The study by Russell et al. reported that no difference in risk was observed between players in forward and backline positions [37]. In a cohort of former Scottish professional Association Football players [43], higher rates of neurodegenerative diseases were associated with longer career durations, but all-cause mortality and neurodegenerative disease mortality were not associated with career duration in a similar study of former French Association Football players [12].

The latest International Consensus Statement on Concussion in Sport [44] and the systematic review informing its section on the potential long-term effects of head injury [10] both emphasised the need for more longitudinal studies in collision sports. Several aspects of the association between participation in high-level rugby and neurodegenerative diseases need further investigation, including whether (1) the higher rates of neurodegenerative disease among rugby players compared to the general population observed in Scotland [37] are similar in other rugby cohorts; (2) the risks of neurodegenerative disease vary by playing positions in rugby; (3) a higher level of play in rugby is a risk factor for developing neurodegenerative disease; and (4) greater exposure to high-level rugby (i.e. the number of years or matches played) influences the risk of neurodegenerative disease. Our study aimed to explore these issues.

## Methods

### Study Design and Participants

This retrospective cohort study is part of the Kumanu Tāngata project, which involves linkage of the New Zealand Rugby Register to Statistics New Zealand’s Integrated Data Infrastructure (IDI), a whole-of-population administrative database that is made available for research under strict access and security conditions [45]. The project has the overarching goal of examining long-term health outcomes of first-class rugby players in New Zealand. This is reflected in the project name, ‘Kumanu Tāngata’, gifted by New Zealand Rugby kaumātua (respected indigenous New Zealand elder) and Māori advisor, the late Luke Crawford, and embodies, in the Māori language, the concept of weaving multiple data threads to support player wellbeing.

Ethics approval was granted by the Auckland Health Research Ethics Committee (ref. AH23203). The study protocol has been previously published [46]. This study followed the Strengthening the Reporting of Observational studies in Epidemiology (STROBE) guidelines [47].

#### Rugby Cohort

Former high-level rugby players were identified using publicly available information from the New Zealand Rugby Register, which contains information on New Zealand rugby players who played at the provincial level or higher (‘first-class rugby’) between 1870 and 2015 [48]. These athletes participated in rugby at the highest level in New Zealand during the study period, in accordance with the definition of first-class rugby from the New Zealand Rugby Union, and are distinct from lower-tier or community-level teams (club, school, and social teams) [49]. Specifically, these players represent an estimated 3–4% of all active male registered Rugby Union players aged 18–30 years, based on contemporary New Zealand Rugby player registration data [50]. Rugby register records for male players who ended their careers no earlier than 1950 and who started their careers no later than 2000 were provided to Statistics New Zealand for linkage to the IDI. Female players were not included in the current study, due to low participation numbers during the study period.

**Fig. 1:**
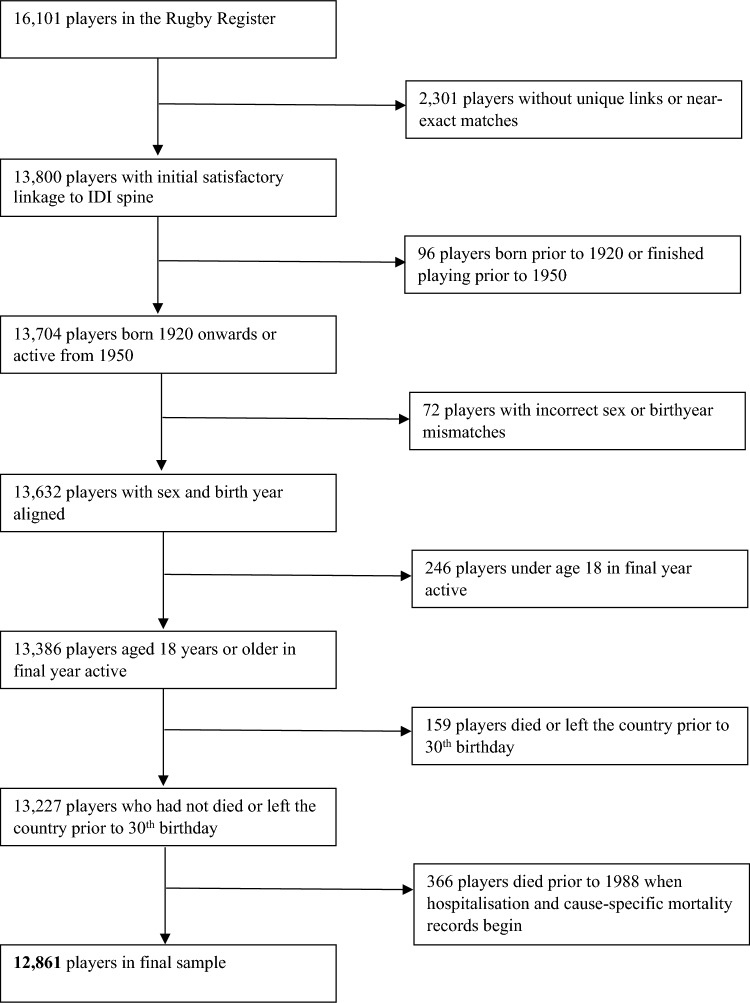
Flowchart demonstrating exclusion criteria applied to players. *IDI* Integrated Data Infrastructure

Exclusion criteria and the number of players excluded for each reason are displayed in the flowchart (Fig. 1) [46]. An in-depth explanation of initial exclusion criteria is provided in the study protocol [46]. Additionally, we excluded individuals who died prior to 1988, when hospitalisation and cause-of-death records became available. We also intended to exclude individuals who developed a neurodegenerative disease before the age of 30, though no individuals in the cohort met this criterion. Of the 16,101 players for whom information was provided to Statistics New Zealand, 12,861 (80%) were included in the analysis cohort.

Player position was categorised into positional groups as either forwards or backs (12%, *n* = 1542 were unable to be coded due to limited positional data). Both career duration (years played) and matches played at the first-class level served as proxy measures for exposure to high-level rugby. Years of play was calculated as last year played minus first year played at the first-class level. These variables reflect participation at the first-class level only, and do not capture exposure to rugby at lower levels.

Highest level of play was categorised as international/professional or provincial/first-class amateur. The former category included players who represented the New Zealand national rugby team (the ‘All Blacks’) since 1950 and those who played in the New Zealand professional ‘Super Rugby’ competition since 1996 (when professional rugby began). The provincial/first-class amateur group included players representing one of 27 provincial rugby teams since 1950 who did not play at the international level or in a professional team.

#### Population Comparison Cohort

A general population comparison group consisted of all males born from 1920 to 1984 (the most recent birth year of the rugby cohort) with a linkable IDI record who were not members of the rugby cohort. Similar exclusion criteria to those for the rugby cohort were applied (see Figure S1 in the electronic supplementary material). This final cohort consisted of 2,394,300 individuals.

### Neurodegenerative Diseases

Neurodegenerative disease diagnoses were identified using International Statistical Classification of Diseases and Related Health Problems 10th Revision, Australian Modification (ICD-10–AM) and corresponding ICD-9 codes in public hospitalisation (National Minimum Dataset) and mortality records from the New Zealand Ministry of Health (see Table S1 in the electronic supplementary material) for the following outcomes: Alzheimer’s disease, other dementias (including ‘unspecified’ dementias), Parkinson’s disease, and motor neuron disease, as well as ‘any neurodegenerative disease’, using a previously developed method [51]. Both primary and contributory cause codes were used. Case identification was based on a combination of hospitalisation and mortality records, with hospitalisation data available from January 1988 to June 2023 and mortality data available from January 1988 to December 2019. This resulted in an overall 35.5-year case-identification period for both players and the general population.

### Statistical Analyses

#### Primary Analyses

To examine whether rugby players had a differential risk of neurodegenerative disease compared to males from the general population, we used Cox proportional hazards models. Participants were tracked from the age of 30 and right censored at death, emigration, or the end of the study period without diagnosis. Schoenfeld residuals tested the proportional hazards assumption. Where this was violated, hazards were computed separately for 10-year age ranges (30–39, 40–49, 50–59, 60–69, 70–79, 80–89 and 90 +), with pre- and post-period events censored. Overall hazard ratios (HRs) reported can be interpreted as the average effect over the entire follow-up period [52].

HRs were estimated for each outcome separately, and the attributable percentage of cases in the rugby group was calculated using 100 × (HR − 1)/HR. The attributable number of cases per year at the end of the case-identification period was determined by multiplying total cases by the attributable percentage and dividing by 35.5 years.

We used direct standardisation to account for birth year (defined in 5-year bands from 1920–24 to 1980–84), ethnicity (European, Māori, Pacific Island, and other [combining Asian, Middle Eastern, Latin American, African, and other ethnicities]), and overseas-born (New Zealand-born, overseas) differences between rugby players and the general population. Weighted descriptives demonstrated near identical sociodemographic compositions after standardisation (Table 1).

**Table 1:** Characteristics of rugby players and general population comparison groups

	Rugby players,* n* (%) *N* = 12,861	Weighted general population,* n* (%) *N* = 2,394,300
Ethnicity		
European	10,371 (80.6)	1,930,929 (80.6)
Māori	2418 (18.8)	449,967 (18.8)
Pacific Island	483 (3.8)	90,105 (3.8)
Asian	39 (0.3)	7446 (0.3)
MELAA	102 (0.8)	18,213 (0.8)
Other	267 (2.1)	40,737 (1.7)
NZ vs overseas born		
NZ born	12,204 (94.9)	2,272,173 (94.9)
Overseas born	657 (5.1)	122,127 (5.1)
Birth year		
1920–1924	357 (2.8)	66,648 (2.8)
1925–1929	927 (7.2)	172,578 (7.2)
1930–1934	1272 (9.9)	236,619 (9.9)
1935–1939	1149 (8.9)	213,720 (8.9)
1940–1944	1215 (9.4)	226,008 (9.4)
1945–1949	1308 (10.2)	243,507 (10.2)
1950–1954	1179 (9.2)	219,678 (9.2)
1955–1959	1116 (8.7)	207,762 (8.7)
1960–1964	1209 (9.4)	225,264 (9.4)
1965–1969	1155 (9.0)	214,653 (9.0)
1970–1974	1143 (8.9)	212,787 (8.9)
1975–1979	753 (5.9)	140,556 (5.9)
1980–1984	78 (0.6)	14,520 (0.6)

*MELAA* Middle Eastern, Latin American, and African, *NZ* New Zealand

Separate Cox models examined the influence of player position, level of play, and exposure to first-class rugby (years/matches played at first-class level) on neurodegenerative disease risk. As the distributions of years played and matches played were highly right-skewed (Figures S2 and S3; see the electronic supplementary material), players were grouped into tertiles representing low, moderate, and high exposure (years played: 1, 2–5, 6 + years; matches played: 1–4, 5–20, 21 + matches). HRs were calculated for player groups compared to the general population. HRs were also calculated for forwards compared to backs, international and/or professional players compared to provincial and/or amateur players, and moderate and high exposure groups compared to the lowest exposure group. As the direct standardisation method accounts for differences between players and the general population, but not for differences between player groups (e.g. by years played, position), covariate adjustment for birth year bands, ethnicity, and New Zealand-born status was used in addition to direct standardisation for these analyses.

#### Secondary Analyses

We further investigated whether associations between player position and neurodegenerative disease risk varied by exposure to high-level rugby, specifically to explore whether players who typically experience greater HAEs (i.e. forwards) [39] and have greater exposure to the sport (i.e. greater cumulative exposure to repeated HAEs) show an increased risk of any neurodegenerative disease. This involved conducting Cox models for years and matches played stratified by player position.

To assess the robustness of primary results, sensitivity analyses were conducted, considering:

- Pharmaceutical prescriptions for neurodegenerative disease treatment. Pharmaceutical data were not used for initial case ascertainment, as records were only available from July 2006. Moreover, pharmaceutical data cannot differentiate between Alzheimer’s disease and other dementias (see Table S1 in the electronic supplementary material).
- Neurodegenerative disease identified through the interRAI, a geriatric assessment administered to older-aged individuals receiving support for at-home care and aged residential care [53]. Data are only available for a minority of the older-aged population from July 2014.
- Competing risks for neurodegenerative disease mortality versus other causes of death.
- The effect of plausible fractions of the general population exposed to rugby as adults at levels below first-class rugby (‘community rugby’) on the observed results, assuming similar levels of neurodegenerative disease risk among first-class and community rugby players.

Additional details for the sensitivity analyses are provided in the electronic supplementary material (see the ‘Sensitivity analyses: methods and data sources’ section).

Reported counts were randomly rounded to a base of 3 in accordance with the confidentiality rules of Statistics New Zealand. Statistical analyses were performed using SAS Enterprise Guide version 8.3 and Stata version 16.1.

## Results

### Comparisons Between Rugby Players and the General Population

The mean follow-up period—from age 30 until a player had a neurodegenerative disease event or was censored—was 37.3 years (SD 13.6; median 37.9 [interquartile range {IQR} 26.5–47.9]) for players and 36.7 years (SD 13.9; median 37.2 [IQR 25.9–47.4]) for the general population. Over this time, 834 (6.5%) of 12,861 players and 124,371 (5.2%) of 2,394,300 males from the general population were identified as having any neurodegenerative disease. The average age at which neurodegenerative conditions were first identified was 79.3 years (SD 8.7; median 80.4 years [IQR 73.9–85.6]) for players and 78.3 (SD 9.4; median 79.7 years [IQR 73.4–84.9]) for the general population.

**Fig. 2:**
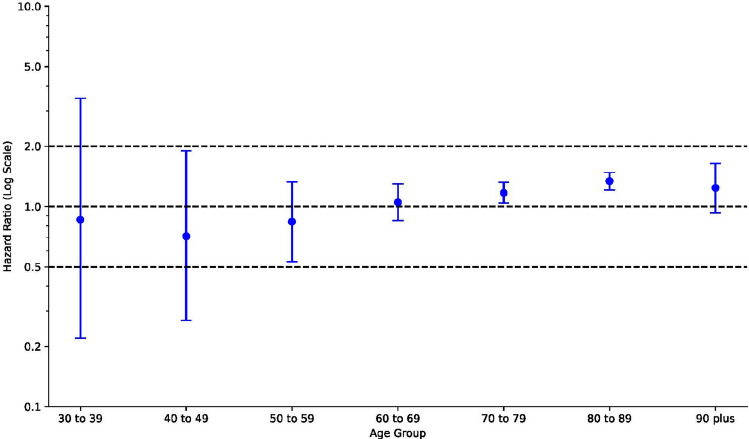
Time-stratified hazard ratios (HRs) for any neurodegenerative disease among players compared to the general population. The age groups represent age ranges during follow-up. All cohort members were followed from age 30, contributing to the HR for the 30–39 age group. Nevertheless, as some cohort members were censored before reaching older ages, progressively fewer contributed to HRs for older age groups

Compared with males from the general population, rugby players were more likely to be diagnosed with any neurodegenerative disease (HR 1.22 [95% CI 1.14–1.30]), Alzheimer’s disease (HR 1.61 [95% CI 1.42–1.83]), and other dementias (HR 1.23 [95% CI 1.14–1.33]), but not with Parkinson’s disease (HR 1.05 [95% CI 0.89–1.22]) or motor neuron disease (HR 1.16 [95% CI 0.83–1.63]) (Table 2). However, the proportional hazards assumption was not met for the ‘any neurodegenerative disease’ and ‘other dementias’ outcomes. Time-stratified analyses revealed that the increased risk of any neurodegenerative disease and other dementias among players compared to the general population did not begin until around 70–79 years of age (Fig. 2; Table S2 in the electronic supplementary material). Kaplan–Meier survival curves for players compared to the general population for each neurodegenerative outcome are provided in supplementary Figures S4–S10.

**Table 2:** Neurodegenerative disease case numbers and hazard ratios in rugby players and New Zealand males

	Cases (percent of group)		Risk difference, percentage points (95% CI)	Hazard ratio (95% CI)^a^	Attributable percentage^b^ in the exposed group	Cases per year given population rate	Cases attributable to being in the rugby group per year given hazard ratio	Average number of cases per year—Jan 1988 to Jun 2023^c^
	Rugby (*n* = 12,861)	General population (*n* = 2,394,300)						
Any neurodegenerative disease^d^	834 (6.5)^e^	124,371 (5.2)	1.29 (0.86–1.72)	1.22 (1.14–1.30)	18 (12–23)	19.3 (18.0–20.7)	4.2 (2.8–5.5)	23.5
Alzheimer’s disease	240 (1.9)	27,603 (1.2)	0.71 (0.48–0.95)	1.61 (1.42–1.83)	38 (29–45)	4.2 (3.7–4.8)	2.6 (2.0–3.1)	6.8
Other dementias^d^	639 (5.0)	93,783 (3.9)	1.05 (0.68–1.43)	1.23 (1.14–1.33)	19 (12–25)	14.6 (13.5–15.8)	3.4 (2.2–4.5)	18.0
Parkinson's disease	159 (1.2)	28,335 (1.2)	0.05 (− 0.14 to 0.24)	1.05 (0.89–1.22)	4 (− 12 to 18)	4.3 (3.7–5.0)	0.2 (− 0.5 to 0.8)	4.5
Motor neuron disease	33 (0.3)	5442 (0.2)	0.03 (− 0.06 to 0.12)	1.16 (0.83–1.63)	14 (− 20 to 39)	0.8 (0.6–1.1)	0.1 (− 0.2 to 0.4)	0.9
Neurodegenerative mortality (primary cause)	201 (1.6)	28,395 (1.2)	0.38 (0.16–0.59)	1.26 (1.10–1.45)	21 (9–31)	4.5 (3.9–5.2)	1.2 (0.5–1.8)	5.7
Neurodegenerative mortality (including contributing cause)	252 (2.0)	34,623 (1.4)	0.51 (0.27–0.75)	1.31 (1.16–1.49)	24 (14–33)	5.4 (4.8–6.1)	1.7 (1.0–2.3)	7.1

*CI* confidence interval

^a^Adjusted for birth year, ethnicity, and born in New Zealand or overseas

^b^Attributable percentage = 100 × (hazard ratio − 1)/hazard ratio

^c^Average number of neurodegenerative cases per year expected among the rugby cohort, given the base rate of conditions in the general population and the hazard ratios comparing the player group to the general population group

^d^Analyses did not meet the proportional hazards assumption and showed time-dependent variability. Time-stratified results are presented in Fig. 2 and Table S2 (see the electronic supplementary material)

^e^As individuals can be diagnosed with more than one type of neurodegenerative disease, the case number for ‘any neurodegenerative disease’ is not the sum of the specific neurodegenerative disease categories

Regarding attributable percentages of players diagnosed with the outcomes of interest, 38% (95% CI 29–45) of former players who had Alzheimer’s disease would not have been expected to have the condition had they not been a member of the group exposed to high-level rugby. The equivalent statistic was 19% (95% CI 12–25) for diagnoses of other dementias and 21% (95% CI 9–31) for mortality from any neurodegenerative disease as the primary cause (Table 2). Given the size of the player cohort and the rates of neurodegenerative diseases identified, 4.2 (95% CI 2.8–5.5) of the 23.5 players per year who developed a neurodegenerative disease by the end of the study would not have been expected to had they not been a member of the group exposed to high-level rugby (Table 2).

### Differences by Playing Position and Level of Play

A total of 6066 players (47.2%) were backs and 5250 (40.8%) were forwards; the remainder could not be classified (*n* = 1542; 12.0%). The mean years played was 5.0 (SD 4.1) for forwards and 4.3 for backs (SD 3.7), and the mean number of matches played was 31.6 for forwards (SD 44.5) and 24.5 (SD 37.2) for backs.

Backs were more likely than the general population to be diagnosed with any neurodegenerative disease (HR 1.30 [95% CI 1.18–1.42]), Alzheimer’s disease (HR 1.84 [95% CI 1.55–2.18]), and other dementias (HR 1.35 [95% CI 1.21–1.50]). However, the assumption of proportional hazards was not met for the ‘any neurodegenerative disease’ outcome. Time-stratified analyses revealed that the increased risk of any neurodegenerative disease for backs compared to the general population began at around 70–79 years of age (Table S2; see the electronic supplementary material). No elevated risk was observed for forwards. Forwards were less likely than backs to be diagnosed with Alzheimer’s disease (HR 0.71 [95% CI 0.52–0.96]) and other dementias (HR 0.81 [95% CI 0.67–0.97]) (Table 3).

**Table 3:** Neurodegenerative disease by player position, compared to general population males

	Cases (% of group)		Hazard ratios (95% CI)		
	Forwards (*n* = 5250)	Backs (*n* = 6066)	Forwards vs general population	Backs vs general population	Forwards vs backs
Any neurodegenerative disease	246 (4.7)	441 (7.3)	1.11 (0.98–1.26)	1.30 (1.18–1.42)^a^	0.87 (0.74–1.02)
Alzheimer's disease	63 (1.2)	135 (2.2)	1.29 (1.00–1.66)	1.84 (1.55–2.18)	0.71 (0.52–0.96)
Other dementias	180 (3.4)	348 (5.7)	1.07 (0.92–1.24)	1.35 (1.21–1.50)	0.81 (0.67–0.97)
Parkinson's disease	54 (1.0)	78 (1.3)	1.07 (0.82–1.40)	1.06 (0.84–1.32)	1.04 (0.72–1.45)
Motor neuron disease	15 (0.3)	12 (0.2)	1.49 (0.90–2.47)	0.87 (0.49–1.53)	1.68 (0.78–3.61)

Counts and percentages for cases in the general population are provided in Table 2. Analyses are adjusted for birth year (5-year groups), overseas-born status, and ethnicity

*CI* confidence interval

^a^Analysis did not meet the proportional hazards assumption and showed time-dependent variability. Time-stratified results are presented in Table S2 (see the electronic supplementary material)

A total of 729 players (5.7%) participated at an international/professional level, and the remaining 12,132 (94.3%) participated at a provincial/first-class amateur level only. Players at the international/professional level and players at the provincial/first-class amateur level both had increased risks of any neurodegenerative disease, Alzheimer’s disease, and other dementias when compared to the general population. For international/professional players, HRs were 1.62 (95% CI 1.18–2.20) for any neurodegenerative disease, 1.97 (95% CI 1.06–3.66) for Alzheimer's disease, and 1.67 (95% CI 1.17–2.37) for other dementias. For provincial/first-class amateur players, HRs were 1.18 (95% CI 1.10–1.26) for any neurodegenerative disease, 1.56 (95% CI 1.37–1.78) for Alzheimer's disease, and 1.19 (95% CI 1.09–1.29) for other dementias. In comparisons between playing levels, international/professional players were found to have increased risks of any neurodegenerative disease (HR 1.42 [95% CI 1.03–1.95]) and other dementias (HR 1.46 [95% CI 1.02–2.10]) compared with provincial/amateur level players (Table 4). The proportional hazards assumption was violated for any neurodegenerative disease and other dementias for provincial/amateur players compared to the general population, with the increased risk among players beginning at around 70–79 years of age (Table S2).

**Table 4:** Neurodegenerative disease occurrence among international and/or professional players, provincial and/or amateur players, and general population males

	Cases (% of group)		Hazard ratios (95% CI)		
	Provincial and/or amateur (*n* = 12,132)	International and/or professional (*n* = 729)	Provincial and/or amateur players vs general population	International and/or professional players vs general population	International and/or professional vs provincial and/or amateur
Any neurodegenerative disease	795 (6.6)	42 (5.8)	1.18 (1.10–1.26)^a^	1.62 (1.18–2.20)	1.42 (1.03–1.95)
Alzheimer's disease	231 (1.9)	12 (1.6)	1.56 (1.37–1.78)	1.97 (1.06–3.66)	1.33 (0.70–2.51)
Other dementias	606 (5.0)	33 (4.5)	1.19 (1.09–1.29)^a^	1.67 (1.17–2.37)	1.46 (1.02–2.10)
Parkinson's disease	150 (1.2)	9 (1.2)	1.02 (0.87–1.19)	1.52 (0.76–3.05)	1.54 (0.75–3.15)
Motor neuron disease	30 (0.2)	s	1.13 (0.80–1.60)	1.91 (0.48–7.63)	1.44 (0.34–6.12)

Counts and percentages for cases in the general population are provided in Table 2. Analyses are adjusted for birth year (5-year groups), overseas born status, and ethnicity

*CI* confidence interval, *s* suppressed by Statistics New Zealand to comply with data privacy regulations

^a^Analysis did not meet the proportional hazards assumption and showed time-dependent variability. Time-stratified results are presented in Table S2 (see the electronic supplementary material)

### Differences by Career Length

The mean years of rugby played at a first-class level was 4.4 (SD 3.8) (median 3 [IQR 1–7]), the mean matches played was 25.6 (SD 39.3) (median 10 [IQR 3–31]), and the mean age of players in their final year active was 26.4 (SD 4.1).

When investigating the effect of years played on neurodegenerative outcomes, players with the greatest exposure to high-level rugby (6 or more years) had an increased risk of any neurodegenerative disease (HR 1.31 [95% CI 1.16–1.47]), Alzheimer’s disease (HR 1.78 [95% CI 1.44–2.20]), other dementias (HR 1.28 [95% CI 1.12–1.47]), and Parkinson’s disease (HR 1.30 [95% CI 1.01–1.67]) when compared to the general population. For players with 2–5 years of participation in high-level rugby, an increased risk was observed for any neurodegenerative disease (HR 1.15 [95% CI 1.02–1.29]), Alzheimer’s disease (HR 1.60 [95% CI 1.29–1.98]), and other dementias (HR 1.17 [95% CI 1.03–1.34]). However, the proportional hazards assumption was not met for any neurodegenerative disease or other dementias. Time-stratified analyses demonstrated that increased risks of any neurodegenerative disease and other dementias were not observed until around 70–79 years of age for players with 2–5 years of participation when compared to the general population (Table S2; see the electronic supplementary material). Players who participated in high-level rugby for 1 year had an increased risk of Alzheimer’s disease (HR 1.37 [95% CI 1.08–1.73]) and other dementias (HR 1.16 [95% CI 1.01–1.33]) (Table 5).

**Table 5:** Neurodegenerative disease by number of years played, compared to general population males

	Cases (% of group)			Hazard ratios (95% CI)				
	1 year (*n* = 4332)	2–5 years (*n* = 4386)	6 + years (*n* = 4143)	1 year vs general population	2–5 years vs general population	6 + years vs general population	2–5 years vs 1 year	6 + years vs 1 year
Any neurodegenerative disease	267 (6.2)	285 (6.5)	285 (6.9)	1.12 (1.00–1.27)	1.15^a^ (1.02–1.29)	1.31 (1.16–1.47)	1.03 (0.87–1.22)	1.18 (0.99–1.40)
Alzheimer’s disease	72 (1.7)	87 (2.0)	84 (2.0)	1.37 (1.08–1.73)	1.60 (1.29–1.98)	1.78 (1.44–2.20)	1.17 (0.85–1.60)	1.34 (0.97–1.84)
Other dementias	210 (4.8)	216 (4.9)	210 (5.1)	1.16 (1.01–1.33)	1.17^a^ (1.03–1.34)	1.28 (1.12–1.47)	1.01 (0.84–1.23)	1.11 (0.92–1.35)
Parkinson’s disease	57 (1.3)	42 (1.0)	60 (1.4)	1.07 (0.83–1.40)	0.77 (0.56–1.04)	1.30 (1.01–1.67)	0.72 (0.48–1.08)	1.24 (0.86–1.79)
Motor neuron disease	9 (0.2)	12 (0.3)	12 (0.3)	0.90 (0.47–1.72)	1.17 (0.66–2.06)	1.44 (0.84–2.48)	1.25 (0.53–2.98)	1.50 (0.63–3.56)

*CI* confidence interval

^a^Analysis did not meet the proportional hazards assumption and showed time-dependent variability. Time-stratified results are presented in Table S2 (see the electronic supplementary material)

In terms of matches played, players in the greatest exposure group (21 or more matches) had an increased risk of any neurodegenerative disease (HR 1.21 [95% CI 1.07–1.37]), Alzheimer’s disease (HR 1.61 [95% CI 1.28–2.03]), other dementias (HR 1.20 [95% CI 1.04–1.39]), and motor neuron disease (HR 1.85 [95% CI 1.15–2.98]) when compared to the general population. Players who participated in 5–20 matches had an increased risk of any neurodegenerative disease (HR 1.29 [95% CI 1.15–1.45]), Alzheimer’s disease (HR 1.77 [95% CI 1.43–2.20]), and other dementias (HR 1.32 [95% CI 1.15–1.50]) compared to general population males. The proportional hazards assumption was violated for any neurodegenerative disease for 5–20 matches, with increased risks among players again beginning at around 70–79 years of age (Table S2). Players who participated in 1–4 matches had an increased risk of Alzheimer’s disease (HR 1.35 [95% CI 1.08–1.69]) relative to the general population (Table 6).

**Table 6:** Neurodegenerative disease by number of matches played, compared to general population males

	Cases (% of group)			Hazard ratios (95% CI)				
	1–4 matches (*n* = 4404)	5–20 matches (*n* = 4026)	21 + matches (*n* = 4302)	1–4 matches vs general population	5–20 matches vs general population	21 + matches vs general population	5–20 matches vs 1–4 matches	21 + matches vs 1–4 matches
Any neurodegenerative disease	282 (6.4)	282 (7.0)	258 (6.0)	1.08 (0.96–1.22)	1.29 (1.15–1.45)^a^	1.21 (1.07–1.37)	1.18 (1.00–1.39)	1.13 (0.95–1.34)
Alzheimer’s disease	78 (1.8)	84 (2.1)	72 (1.7)	1.35 (1.08–1.69)	1.77 (1.43–2.20)	1.61 (1.28–2.03)	1.28 (0.94–1.74)	1.19 (0.86–1.64)
Other dementias	216 (4.9)	216 (5.4)	195 (4.5)	1.10 (0.97–1.26)	1.32 (1.15–1.50)	1.20 (1.04–1.39)	1.19 (0.98–1.43)	1.10 (0.91–1.34)
Parkinson’s disease	57 (1.3)	48 (1.2)	48 (1.1)	1.00 (0.77–1.30)	1.05 (0.80–1.39)	1.03 (0.78–1.37)	1.04 (0.71–1.52)	1.04 (0.71–1.53)
Motor neuron disease	9 (0.2)	9 (0.2)	18 (0.4)	0.84 (0.44–1.61)	0.88 (0.44–1.75)	1.85 (1.15–2.98)	1.01 (0.39–2.63)	2.07 (0.92–4.68)

Analyses are adjusted for birth year (5-year groups), overseas born status, and ethnicity

*CI* confidence interval

^**a**^Analysis did not meet the proportional hazards assumption and showed time-dependent variability. Time-stratified results are presented in Table S2 (see the electronic supplementary material)

### Secondary Analyses

Associations between exposure and any neurodegenerative disease stratified by position group are shown in Table S3 and Figure S11 (see the electronic supplementary material). While rates of neurodegenerative diseases increased with greater exposure for backs, they did not for forwards. Specifically, for backs compared to the general population, HRs were 1.38 (95% CI 1.18–1.62) for participation in 5–20 matches, 1.42 (95% CI 1.21–1.68) for 21 or more matches, and 1.59 (95% CI 1.36–1.86) for 6 or more years of play.

Sensitivity analyses including pharmaceutical data and data from a geriatric assessment (interRAI) showed that results remained similar (Tables S4 and S5). Analyses accounting for non-neurodegenerative disease mortality as a competing risk showed minimal impact on results (Table S6). Similarly, varying the assumed proportions of lower-level (community) rugby players in the general population showed only minor changes in the observed effects (Table S7).

## Discussion

We found a small increased risk of neurodegenerative disease diagnoses among male high-level rugby players compared to males from the general population in New Zealand. Further, we observed a time-dependent relationship between rugby and neurodegenerative disease, in which the increased risk among players generally did not begin until around 70–79 years of age. Backline, provincial/amateur level and international/professional players showed greater risks of neurodegenerative disease compared to the general population. We also found an increased risk of neurodegenerative disease for players with moderate-to-high levels of exposure to rugby (years and matches played) when compared to the general population. However, while we found some evidence of a dose–response association between participation in high-level rugby and neurodegenerative outcomes, clear conclusions on a dose–response relationship could not be reached as associations varied across outcomes.

From a public health perspective, both the population prevalence of a condition and the change in that prevalence when individuals are exposed to a presumed cause are important considerations. We observed that out of every 1000 men in the general population, 52 died from or were diagnosed with a neurodegenerative disease over the follow-up period. Among former rugby players, the number was 65 per 1000; i.e. an extra 13 cases per 1000 people over the study period, or approximately four extra neurodegenerative disease cases per year, given the size of the player cohort.

The effects observed in our study are generally in the same direction, albeit smaller, than those reported in a recent study of neurodegenerative diseases amongst former international rugby players from Scotland [37]. In the Scottish study, there was a 167% (95% CI 67–327) higher rate of incident neurodegenerative disease among former international players than the general population, whereas in our study the corresponding statistic for international and/or professional players was 62% (95% CI 18–120). Our results are consistent with previous research showing elevated risks of neurodegenerative disease mortality in former high-level athletes from a range of collision sports, although again the HRs and associated statistics observed in our study were lower than those previously reported [9, 11–14]. For example, among former players in our study who died from any neurodegenerative disease, the percentage of deaths attributable to being a member of the rugby group was 24% as a primary or contributory cause (21% as a primary cause of death only). In contrast, for Scottish rugby players, it was 62% [37]; for Association Football players from Sweden [13], Scotland [11], and France (primary cause of death only) [12], the attributable percentages were 35%, 72%, and 60% respectively; and for American Football players, it was 69% [14].

Differences in effect size observed across the studies could be due to several factors, such as differences in linkage rates, the composition of the comparison group, sample size, covariates measured, age and time period of investigation, case identification processes, and competition differences in typical player exposure to certain activities (e.g. contact events) [12]. Many of the aforementioned studies also focused on a professional or elite-level athlete cohort, whereas our cohort includes those who participated at a lower (albeit still first-class) level. Overall, these differences in magnitude highlight the need for further investigation into the neurodegenerative disease outcomes associated with participation in contact sports using robust study designs.

Existing research on the association between playing position in contact/collision sports and the risk of neurodegenerative diseases is equivocal. While findings from soccer suggest that the accumulation of HAEs plays an important role in the risk of neurodegenerative outcomes, with positions exposed to more HAEs showing an association with neurodegenerative disease [11, 13], results from rugby and American Football are less clear. In the current study, we found that players in forward positions were less likely to be diagnosed with Alzheimer’s disease and other dementias than players in back positions. Moreover, while backs were more likely to be diagnosed with any neurodegenerative disease, Alzheimer’s disease, and other dementias when compared to the general population, there were no differences observed between forwards and the general population. This suggests that the increased risk of neurodegenerative disease among rugby players may largely be driven by a greater risk amongst players in back positions.

Our results are consistent with those of previous research in American Football in showing a greater risk of neurodegenerative disease among players in speed positions (e.g. backs) than players in non-speed positions (e.g. forwards) [14]. Given evidence in both rugby and American Football that non-speed players are typically exposed to a greater number of HAEs than speed players [14, 38, 39], these findings do not appear to support the hypothesis that increases in neurodegenerative disease rates among former rugby players are primarily driven by cumulative exposure to repetitive HAEs. Additionally, there was no apparent increase in neurodegenerative disease rates associated with more years or matches played for forwards, which would be expected if cumulative exposure to repeated HAEs were mainly responsible for the development of neurodegenerative diseases among players. In contrast, this association was present for backs. This implies that the nature of contact events (i.e. exposure to high-speed collisions), and not just exposure to repetitive HAEs, may influence neurodegenerative disease risk in contact sport athletes. Overall, the literature regarding positional findings remains mixed, with some studies of American Football and rugby finding no association between playing position and neurodegenerative disease [37, 54]. It remains unclear whether it is predominantly the nature or the frequency of HAEs that influences the risk of neurodegenerative disease outcomes in contact sports. There is a need for further research to elucidate the mechanisms underlying the association between participation in contact sports and neurodegenerative disease risk, and the sport-specific factors that may influence this risk.

We observed that both provincial and/or amateur players and international and/or professional rugby players in our cohort had a small-to-moderate increased risk of any neurodegenerative disease, Alzheimer’s disease, and other dementias when compared to the general population, though the overall effect was greater for those who participated at international and/or professional-level play. This difference may partly reflect the greater number of years and matches played by international and/or professional players, as they typically progress through years of provincial rugby before achieving the level required for international selection, resulting in a higher cumulative exposure to the sport. Ideally, our analysis would have further distinguished professional players as a separate cohort, but this group was aggregated with international players by Statistics New Zealand to maintain data confidentiality.

Our results also indicate that, overall, the risk for Parkinson’s disease showed no clear association with high-level rugby, with only minimal evidence suggesting risks may be increased for players with 6 or more years of exposure. Additionally, while the results for motor neuron disease showed slightly more evidence of an association with high-level rugby, particularly for 21 or more matches, CIs were generally wide and almost all included unity. Motor neuron disease is relatively rare, making it challenging to conduct epidemiological research with sufficiently large numbers of cases to permit robust inferences about postulated causes. While a systematic review and meta-analysis published in 2023 concluded that professional athletes exposed to sports such as football or soccer have an increased risk of developing motor neuron disease, the comparative lack of an increase for motor neuron disease we observed in players overall compared to the general population aligns more closely with findings from studies of non-professional footballers from various codes [10]. However, as we did observe an increased risk of motor neuron disease associated with a higher number of matches played, this raises questions about the effects of exposure to greater levels of environmental factors in field-based sports (e.g. pesticides or soil-borne pathogens) [18, 19], as well as greater exposure to repeated HAEs [55] as potential contributors.

Several hypotheses have been proposed to explain the mechanisms underlying the increased risk of neurodegenerative diseases observed among collision sport athletes more broadly:

1. The excess risk is primarily due to CTE-NC, which represents a neurodegenerative disease with a progressive course that is caused by repeated HAEs, and that results in dementia [15].
2. Dementia occurs in the presence of CTE-NC along with co-morbid neuropathologies, possibly due to neuroinflammatory responses from repeated HAEs [16, 17].
3. Exposure to repeated HAEs might decrease ‘cognitive or cerebral reserve’, lowering the age at which an existing neurodegenerative disease becomes symptomatic and resulting in a greater number of individuals experiencing neurodegenerative outcomes [6].

However, in our cohort, the increased risk of neurodegenerative diseases was observed from age 70 onwards, indicating no evidence of an accelerated or early-onset disease course. This finding contrasts with established literature linking single moderate-to-severe TBIs to an earlier onset of dementia [22, 56, 57]. While our study was not designed to test specific mechanisms, the higher risk later in life suggests two main hypotheses. First, the pathological pathway associated with cumulative exposure to HAEs over a career may differ from that of a single severe TBI, potentially by lowering the threshold for age-related neurodegeneration rather than accelerating its clinical onset. Second, and consistent with the 'healthy worker effect', the high baseline health and cognitive reserve required for selection into high-level sport may provide a protective buffer that delays the clinical expression of disease. These hypotheses are not mutually exclusive and underscore the need for future prospective studies that directly measure head impact exposure alongside other potential mediating and confounding factors. Moreover, our positional findings suggest that other factors, such as the nature of contact events associated with brain trauma in combination with extended playing careers, may also play a role.

Beyond brain trauma, the relative contributions of factors that high-level collision sport athletes are collectively differentially exposed to when compared to the wider population from which they are drawn requires further investigation [58]. Prospective follow-up studies using large player cohorts and comprehensive data collection (including frequency and nature of HAEs, personal, sport-related, and lifestyle factors, and post-mortem evaluations) are the next step to better understand underlying mechanisms. However, as the results of prospective follow-up studies examining late-life health outcomes take a generation to emerge, we recommend that, in the interim, collision sports organisations act in accordance with the precautionary principle [59] to limit exposure to repeated HAEs, proactively manage suspected concussions, and clearly communicate to the public what is understood about both the benefits and the risks of participation in the sport they administer.

### Strengths and Limitations

Our study has notable strengths. To limit confounding due to demographic differences, we used a comprehensive population database to create a large cohort of first-class male New Zealand rugby players alongside a comparison group of New Zealand males statistically weighted to align with the demographic traits of the players. The data available also permitted sensitivity analyses that increase confidence in our findings, including the addition of prescription medications for case identification and accounting for non-neurodegenerative causes of death. The study had a lengthy follow-up period, enabling the assessment of neurodegenerative disease outcomes with substantial data. The large cohorts and extended follow-up period contributed to more precise effect estimates than prior research on rugby players.

However, our study does have limitations. As with other retrospective studies of former collision sport athletes, there may have been unmeasured differences in health-related attributes and behaviours between the player group and the general population both prior to and after their exposure to collision sports. This includes an inability to accurately account for a prior history of brain trauma (from any cause), other potential risk factors for dementia (e.g. education, alcohol use, smoking, diabetes) [1], and confounders (e.g. socioeconomic status [SES]), which have been addressed in similar studies [37]. For instance, alcohol use, smoking, and education level are not measured well in the administrative data used for this study (the IDI), particularly for the ages we investigated. SES data are only available in the IDI from 1997 onwards, which is the post-career period for most of the players in our cohort, and may therefore reflect an outcome of first-class rugby participation rather than a pre-exposure confounder. However, prior studies that adjusted for a wide range of risk factors still observed an increased risk of neurodegenerative disease amongst former contact sports athletes. For example, Batty et al. [9] noted that adjustments for various risk factors (i.e. SES, physical health comorbidities, smoking and alcohol behaviour, and body mass index) had little impact on the magnitude of effects.

In the current study, the player group and the associated information we were able to obtain (e.g. years and matches played) were limited to participation at first-class level and above, though it is likely that these players had substantial exposure to rugby at lower levels of play prior to, and during, their involvement at a higher level [42]. Female players were not included due to the low number of female players during the period studied. Thus, the findings might not apply to lower-level male players or to female players. Rapidly increasing female rugby player participation warrants similar research of this cohort in time.

It is also important to note that the first year played among the playing cohort included only 5 years in which rugby was a professional sport (1996–2000). Therefore, the changes associated with professionalism—such as increased training volumes, larger players, and more frequent collisions [60]—and the potential effects on neurodegenerative risk, combined with demographic changes (e.g. a higher proportion of high-level rugby players from Pacific ethnicities), mean that the study findings may not generalise to high-level players who began their career after 2000. Equally, modern professional players will have been managed under head injury assessment protocols introduced in the past decade, representing a shift from earlier concussion management approaches. Most players who participated in the 1990s, when professional rugby began, would currently be aged 45–65, meaning that health outcomes that typically manifest in later life, such as Alzheimer’s disease, would not yet have developed in most cases.

The popularity of rugby in New Zealand during the twentieth century implies that the general population cohort likely comprises individuals with various degrees of rugby involvement, though data on such involvement were not available. Exposure to collision sports among the general population could potentially attenuate the observed differences between high-level players and the general population, although sensitivity analyses indicate that the effects of community rugby players in the general population on the results obtained are likely small (Table S7; see the electronic supplementary material).

Challenges in documenting causes of death via death certificates present further limitations. A review revealed substantial undercounting of dementia-related deaths in death certificate data, leading to underestimated dementia rates [61]. We addressed these issues by using data from hospitalisation records in addition to death certificate data, along with a sensitivity check provided by using additional sources for identifying neurodegenerative diseases—pharmaceutical prescriptions and a geriatric assessment—which showed comparable results.

It is possible that dementia was undercounted both in players and the general population due to low diagnostic coverage for dementia and the fact that primary care consultation data are currently not available within the IDI [51]. It is also worth noting that while neurodegenerative disease diagnoses were obtained from official hospitalisations and mortality records, only a limited number of individuals undergo autopsy to definitively identify neuropathologies. Nevertheless, we believe that systematic discrepancies in death certificate coding between players and the general population are improbable, so although the neurodegenerative disease estimates might be conservative, the relative rates presented are good estimates of the population effects.

## Conclusions and Future Research

The current study showed that former male high-level rugby union players in New Zealand have an increased risk of neurodegenerative disease when compared to males from the general population. This increased risk generally began at around 70–79 years of age. Small to moderate increased risks of any neurodegenerative disease were observed for a backline playing position, provincial and/or amateur players, international and/or professional players, and participation in ≥ 2 years of play and five or more matches. Our findings also suggest that while the accumulation of brain trauma may be a key contributor to associations between collision sports and neurodegenerative disease, other factors such as the nature of contact events may also play a crucial role. Further research is needed to elucidate the mechanisms underlying the association between contact/collision sports and neurodegenerative disease and to investigate other lifelong health outcomes among this population (e.g. cardiovascular disease, cancer). It remains to be seen whether findings from the current study are applicable to female players, players at lower levels, and contemporary rugby.

## Supplementary Information

Below is the link to the electronic supplementary material.

Supplementary file1 (DOCX 549 KB)

## Declarations

### Availability of data and material

The data analysed in this study were housed in the New Zealand IDI, which is administered by Statistics New Zealand. Anonymised individual-level data in the IDI can only be accessed via secure data laboratories, and only export of approved aggregated results, as presented herein, is permitted under New Zealand privacy regulations. Permission to access data is only granted upon application to Statistics New Zealand as part of bona fide research projects. For these reasons, data cannot be made generally available. Note, these results are not official statistics. They have been created for research purposes from the IDI, which is carefully managed by Statistics New Zealand. For more information about the IDI, please visit https://www.stats.govt.nz/integrated-data/. Access to the data was approved under project MAA2016-21.

### Code availability

Statistical code for analyses can be made available from the corresponding author upon request.
