# Supplementary material for: Neurodegenerative Diseases in Male Former First-Class New Zealand Rugby Players
Source: Sports Med. 2025 Sep 4;56(2):543–58. doi: 10.1007/s40279-025-02299-y (PMC12982275; doi:10.1007/s40279-025-02299-y)
Supplement: Supplementary file 1 — Supplementary file1 (DOCX 549 KB) [file 40279_2025_2299_MOESM1_ESM.docx]

Neurodegenerative diseases in male former first-class New Zealand rugby players: Kumanu Tāngata—the aftermatch project

Francesca Anns, BA(Hons), Kenneth L. Quarrie, PhD, Barry J. Milne, PhD, Chao Li, PhD, Andrew J. Gardner, PhD, Ian R. Murphy, MB ChB, Evert Verhagen, PhD, Craig Wright, BSc, Susan M. B. Morton, PhD, Thomas Lumley, PhD, Lynette Tippett, PhD, Stephanie D’Souza, PhD

**Corresponding Author**

Dr Stephanie D’Souza

Centre of Methods and Policy Application in the Social Sciences, University of Auckland, Auckland, New Zealand

School of Social Sciences, University of Auckland, Auckland, New Zealand

Email: [s.dsouza@auckland.ac.nz](mailto:s.dsouza@auckland.ac.nz)

# Supplementary Material

## Sensitivity analyses: methods and data sources

The Cox-proportional hazard models were replicated for players compared to the general population with the inclusion of pharmaceutical data (in addition to hospitalisations and mortality data) in the classification of neurodegenerative disease outcomes.

Pharmaceutical data are maintained by the NZ Pharmaceutical Management Agency (PHARMAC) for medications subsidised by the New Zealand government (chemical IDs shown below in Table S1).

For the sensitivity analyses including pharmaceutical prescriptions data shown in Table S4, Alzheimer’s disease and other dementias were combined into one ‘dementias’ category due to some conditions being prescribed the same medications (e.g., Alzheimer’s disease and Parkinson’s disease dementia). Pharmaceutical records were available from July 2006 to December 2022.

For the sensitivity analyses including the interRAI data shown in Table S5, only the overall association with ‘any neurodegenerative disease’ was computed. The interRAI is a comprehensive geriatric health assessment given to individuals receiving support for at-home care and aged residential care. The assessment is given to <10% of those aged 60-69, and around 25% of those aged 70-79, 45% of those aged 80-89, and 29% of those aged 90+ [1]. Those whose interRAI assessment indicated they had “Alzheimer’s Disease”, “Other Dementia” or ‘Parkinson’s Disease” were included in the ‘any neurodegenerative disease’ category. InterRAI data were available from July 2014 to June 2023.

Competing risks analyses accounting for non-neurodegenerative disease mortality were also undertaken for the comparisons between the players and the general population as an additional sensitivity check (Table S6). A further sensitivity analysis examined the effect on the observed hazard ratios under a range of plausible fractions of the general population exposed to rugby as adults at community rugby level over the study period on the observed results, assuming similar levels of neurodegenerative disease risk among first-class and community rugby players (Table S7).

| **Neurodegenerative disease** | **ICD-10 codes** | **ICD-9 codes** | **PHARMAC chemical IDs (description)** |
| --- | --- | --- | --- |
| Alzheimer’s disease | F00, F00.0, F00.1, F00.2, F00.9, G30, G30.0, G30.1, G30.8, G30.9 | 290.0, 331.0 | Donepezil (392325, 392326)  Rivastigmine (403725, 403726) |
| Other dementias | F01, F01.0, F01.1, F01.2, F01.3, F01.8, F01.9, F02, F02.0, F02.1, F02.2, F02.3, F02.4, F02.8, F03, F05.1, F10.7, F13.7, F18.7, F19.7, G31.0, G31.1, G31.3 | 290.3, 290.8, 290.9, 290.10, 290.11, 290.12, 290.13, 290.20, 290.21, 290.40, 290.41, 290.42, 290.43, 291.2, 292.82, 294.1, 331.1, 331.2 |  |
| Parkinson’s disease | G20, G21.0, G21.1, G21.2, G21.3, G21.4, G21.8, G21.9, G22 | 332.0, 333.92, 332.11, 332.12, 332.13, 332.18, 332.19 | Dopamine receptor agonists: apomorphine (102425 102426), ropinirole (383025, 383026, 383027, 383028)    Levodopa: levodopa + benserazide (171702, 171706, 171707, 171708, 171709), levodopa + carbidopa (172301, 172302, 172303, 172325)    Monoamine-oxidase-B inhibitors: selegiline (264201)    Catechol-O-methyltransferase inhibitors: entacapone (382925), tolcapone (113801) |
| Motor neurone disease | G12.2 | 335.2, 335.20, 335.21, 335.22, 335.23, 335.24, 335.29 | Riluzole (401725) |

*Note:* ICD-10-AM and ICD-9-CMA-II codes were used for neurodegenerative disease identification in public-hospitalisations and mortality data. ICD-9-CMA-II codes were used for records prior to July 1999. ICD-10-AM codes for Alzheimer’s and other dementias were based on Walesby et al. [2], ICD-9-CMA-II codes for AD and other dementias were based on Richmond-Rakerd et al. [3], Parkinson’s disease codes were obtained from Pitcher et al. [4], and ICD-10-Am codes for MND were obtained from Cao et al. [5], with back mapping to ICD-9-CMA-II codes conducted using masterb10 file from: <https://www.health.govt.nz/nz-health-statistics/data-references/mapping-tools/mapping-between-icd-10-and-icd-9>.

# Table S1. Neurodegenerative disease ascertainment codes from public-hospital, mortality, and pharmaceutical records

|  | **Age group (years) for hazard ratios (95% CI)** | | | | | | |
| --- | --- | --- | --- | --- | --- | --- | --- |
|  | **30-40** | **40-50** | **50-60** | **60-70** | **70-80** | **80-90** | **90+** |
| **Players vs general population** | | | | | | | |
| Any neurodegenerative disease | 0.86 (0.22–3.47) | 0.71 (0.27–1.90) | 0.84 (0.53–1.33) | 1.05 (0.85–1.30) | 1.17 (1.04–1.32) | 1.34 (1.21–1.48) | 1.24 (0.93–1.64) |
| Other dementias | --^a^ | 0.84 (0.21–3.38) | 0.57 (0.26–1.27) | 0.94 (0.69–1.26) | 1.21 (1.05–1.38) | 1.32 (1.18–1.47) | 1.26 (0.94–1.68) |
| **Years played (vs general population)** | | | | | | | |
| Any neurodegenerative disease  - 2-5 years | 1.30 (0.18–9.28) | 1.59 (0.51–4.93) | 0.67 (0.28–1.61) | 0.71 (0.46–1.10) | 1.15 (0.94–1.41) | 1.30 (1.10–1.54) | 1.44 (0.92–2.26) |
| Other dementias   – 2-5 years | --^a^ | 1.25 (0.18–8.91) | 0.82 (0.27–2.55) | 0.75 (0.43–1.33) | 1.11 (0.87–1.41) | 1.31 (1.09–1.57) | 1.43 (0.89–2.30) |
| **Games played vs general population** | | | | | | | |
| Any neurodegenerative disease  – 5-20 matches | --^a^ | --^a^ | 1.18 (0.59–2.36) | 0.70 (0.44–1.12) | 1.28 (1.05–1.57) | 1.57 (1.33–1.85) | 1.06 (0.63–1.79) |
| **Player position vs general population** | | | | | | | |
| Any neurodegenerative disease  – backs | --^a^ | 1.13 (0.36–3.51) | 1.07 (0.59–1.93) | 0.98 (0.71–1.35) | 1.29 (1.10–1.51) | 1.51 (1.32–1.73) | 1.25 (0.85–1.83) |
| **Procode vs general population** | | | | | | | |
| Any neurodegenerative disease  – Provincial and/or amateur | 0.46 (0.07–3.31) | 0.75 (0.28–2.01) | 0.87 (0.55–1.38) | 1.06 (0.86–1.32) | 1.14 (1.01–1.29) | 1.31 (1.18–1.45) | 1.04 (0.77–1.40) |
| Other dementias   – Provincial and/or amateur | --^a^ | 0.89 (0.22–3.57) | 0.59 (0.27–1.32) | 0.94 (0.70–1.28) | 1.19 (1.03–1.36) | 1.29 (1.15–1.44) | 1.05 (0.77–1.43) |

*Note:* CI = Confidence Interval. ^a^Unable to be computed because of low case numbers

**Table S2.** Hazards ratios stratified by follow-up age for analyses that did not meet the proportional hazards assumption

|  | **Hazard ratio (95% CI)** | | | | | | | | | |
| --- | --- | --- | --- | --- | --- | --- | --- | --- | --- | --- |
|  | **Years played** | | | | | **Matches played** | | | | |
| **Playing position** | **1 year vs general population** | **2–5 years vs general population** | **6+ years vs general population** | **2–5 years vs 1 year** | **6+ years vs 1 year** | **1–4 vs general population** | **5–20 vs general population** | **21+ vs general population** | **5**–**20 vs 1**–**4** | **21+ vs 1**–**4** |
|  |  |  |  |  |  |  |  |  |  |  |
| Forwards | 1.22  (0.94–1.58) | 1.09  (0.89–1.35) | 1.07  (0.88–1.31) | 0.89  (0.64–1.24) | 0.88  (0.63–1.22) | 1.08  (0.83–1.41) | 1.22  (1.00–1.49) | 1.04  (0.85–1.28) | 1.09  (0.78–1.53) | 0.96  (0.69–1.35) |
|  |  |  |  |  |  |  |  |  |  |  |
| Backs | 1.11  (0.92–1.32) | 1.22  (1.05–1.43) | 1.59  (1.36–1.86) | 1.12  (0.88–1.42) | 1.48  (1.17–1.89) | 1.10  (0.93–1.30) | 1.38  (1.18–1.62) | 1.42  (1.21–1.68) | 1.28  (1.01–1.61) | 1.34  (1.05–1.69) |
|  |  |  |  |  |  |  |  |  |  |  |

*Note:* CI = Confidence Interval.

**Table S3.** Hazards ratios for any neurodegenerative disease by exposure and playing position

##

|  | **Cases (% of group)** | | **Hazard ratio (95% CI)** | **Attributable percentage in the exposed group** |
| --- | --- | --- | --- | --- |
| **Outcome** | **Rugby**  **(n = 12,861)** | **General population**  **(n = 2,394,252)** |  |  |
| Any neurodegenerative disease | 1,023 (8.0) | 154,086 (6.4) | 1.21 (1.13–1.28) | 17 (12–22) |
| Other dementias | 828 (6.4) | 114,645 (4.8) | 1.31 (1.22–1.40) | 24 (18–29) |
| Parkinson’s disease | 279 (2.2) | 51,918 (2.2) | 1.01 (0.90–1.13) | 1 (-11–12) |
| Motor neuron disease | 36 (0.3) | 5,529 (0.2) | 1.15 (0.82–1.61) | 13 (-22–38) |

*Note:* CI = Confidence Interval. Attributable percentage = 100* (Hazard ratio – 1)/Hazard ratio. Case numbers are random rounded to base 3 to comply with Statistics NZ regulations on data privacy. In line with the exclusion criteria for the primary analyses, individuals who were dispensed a neurodegenerative disease-related pharmaceutical prior to their 30^th^ birthday were excluded from analyses (n = 48, all from the general population). As such, the general population was reweighted for these analyses.

**Table S4.** Sensitivity analysis of neurodegenerative disease prevalence (deaths, hospitalisations, and pharmaceutical prescriptions) and hazard ratios for neurodegenerative disease subtypes in rugby players and comparable New Zealand males

|  | **Cases (% of group)** | | **Hazard ratio (95% CI)** | **Attributable percentage in the exposed group** |
| --- | --- | --- | --- | --- |
| **Outcome** | **Rugby**  **(n = 12,861)** | **General population**  **(n = 2,394,252)** |  |  |
| Any neurodegenerative disease | 1,107 (8.6) | 167,262 (7.0) | 1.21 (1.14–1.28) | 17 (12–22) |

*Note:* CI = Confidence Interval. Attributable percentage = 100* (Hazard ratio – 1)/Hazard ratio. Case numbers are random rounded to base 3 to comply with Statistics NZ regulations on data privacy. In line with the exclusion criteria for the primary analyses, individuals who were dispensed a neurodegenerative disease-related pharmaceutical prior to their 30^th^ birthday were excluded from analyses (n = 48, all from the general population). As such, the general population was reweighted for these analyses.

**Table S5.** Sensitivity analysis of neurodegenerative disease prevalence (deaths, hospitalisations, pharmaceutical prescriptions, and interRAI assessment) and hazard ratio for any neurodegenerative disease in rugby players and comparable New Zealand males

| **Outcome** | **Sub-hazard ratio (95% CI)** |
| --- | --- |
| Any neurodegenerative disease | 1.26 (1.17–1.34) |
| Alzheimer’s disease | 1.65 (1.46–1.88) |
| Other dementias | 1.27 (1.18–1.38) |
| Parkinson’s disease | 1.08 (0.90–1.29) |
| Motor neuron disease | 1.20 (0.86–1.68) |
| Neurodegenerative mortality (primary cause) | 1.30 (1.11–1.53) |
| Neurodegenerative mortality (including contributing cause) | 1.35 (1.20–1.53) |

*Note:* CI = Confidence Interval.

Table S6. Competing risks for non-neurodegenerative disease causes of death for players compared to general population on neurodegenerative disease hospitalisation and mortality.

|  | **Hazard ratios (95% CI) if community level players have the same NDD rates as the higher-level players and the general population consists of:** | | | | |
| --- | --- | --- | --- | --- | --- |
| **Condition** | **As analysed - ignoring community players** | **5% of community rugby players** | **10% of community rugby players** | **15% of community rugby players** | **20% of community rugby players** |
| Any neurodegenerative disease | 1.22 (1.14–1.30) | 1.23 (1.14–1.32) | 1.24 (1.15–1.34) | 1.26 (1.16–1.36) | 1.28 (1.17–1.39) |
| Alzheimer’s disease | 1.61 (1.42–1.83) | 1.65 (1.44–1.89) | 1.70 (1.47–1.96) | 1.75 (1.51–2.04) | 1.81 (1.55–2.13) |
| Other dementias | 1.23 (1.14–1.33) | 1.25 (1.15–1.36) | 1.26 (1.16–1.38) | 1.28 (1.17–1.40) | 1.30 (1.18–1.43) |
| Parkinson's disease | 1.05 (0.89–1.22) | 1.05 (0.89–1.24) | 1.05 (0.88–1.25) | 1.05 (0.88–1.27) | 1.06 (0.87–1.29) |
| Motor neuron disease | 1.16 (0.83–1.63) | 1.17 (0.82–1.67) | 1.18 (0.81–1.72) | 1.19 (0.80–1.78) | 1.21 (0.79–1.84) |
| Neurodegenerative mortality (main cause) | 1.26 (1.10–1.45) | 1.28 (1.10–1.48) | 1.30 (1.11–1.51) | 1.32 (1.12–1.55) | 1.34 (1.12–1.59) |
| Neurodegenerative mortality (contributing cause) | 1.31 (1.16–1.49) | 1.33 (1.17–1.52) | 1.35 (1.18–1.56) | 1.38 (1.19–1.60) | 1.41 (1.20–1.64) |

*Note:* CI = Confidence Interval. NDD = Neurodegenerative disease. The number of adult male rugby players in New Zealand between 2005 and 2017 represented approximately 5% of the adult male population aged between18 and 40. The other fractions are provided because a greater fraction of New Zealand males may have been involved in adult rugby during the period 1950-1980 than has been the case since. The adjustment to the hazard ratio in the ‘As analysed’ column for each of the other percentages of community players was: HR^(1/(1-percent of community players)).

# Table S7. Sensitivity analysis of the hazard ratios under a range of community rugby participation rates in which rugby players at community level are assumed to have similar neurodegenerative disease outcomes to those at higher levels

44,145 died prior to 1988 when hospitalisation and cause-specific mortality records begin

2,973,852 males not involved in high-level rugby

14,097 males included in the rugby register as high-level players or referees

283,107 males died or left the country prior to 30^th^ birthday

2,690,745 males who had not died or left the country prior to 30^th^ birthday

252,261 males did not share characteristics with any rugby player

2,438,484 males who share demographic characteristics with rugby players

2,987,949 males in the general population born between 1920–1984

2,394,339 alive in1988

39 hospitalised with or died from a neurodegenerative disease prior to 30^th^ birthday

**2,394,300** males in the final general population sample

# Fig. S1 Flowchart demonstrating exclusion criteria applied to males from the general population


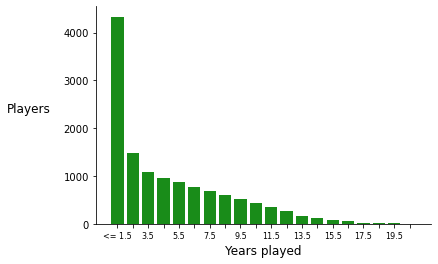


# Fig. S2 Histogram of years played by number of players


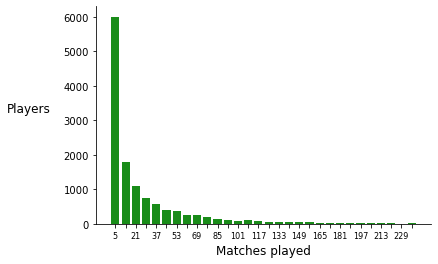


# Fig. S3 Histogram of matches played by number of players

**Kaplan-Meier survival curves for players compared to the general population**


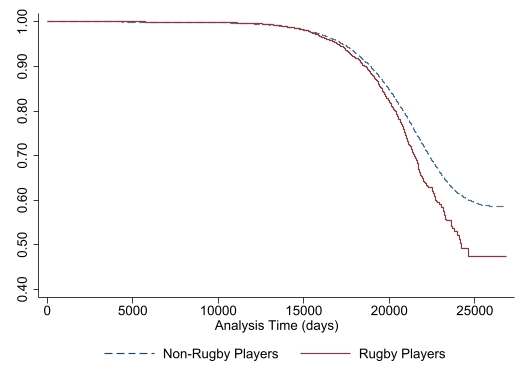


**Fig. S4** Kaplan-Meier survival estimates for any neurodegenerative disease for rugby players compared to the general population


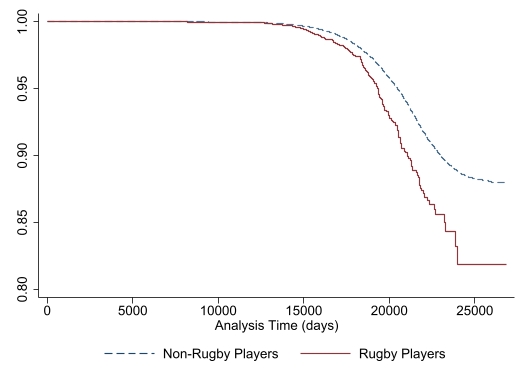


**Fig. S5** Kaplan-Meier survival estimates for Alzheimer’s disease for rugby players compared to the general population


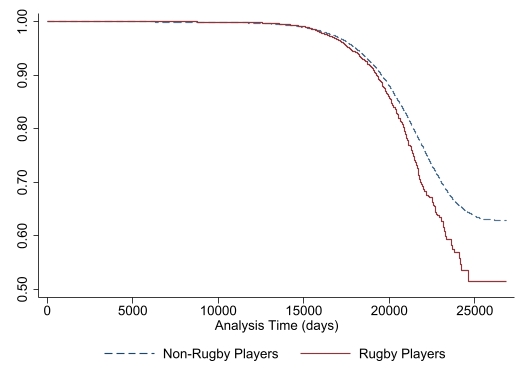


**Fig. S6** Kaplan-Meier survival estimates for other dementias for rugby players compared to the general population


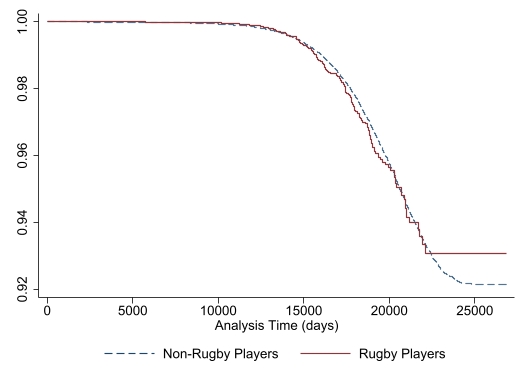


**Fig. S7** Kaplan-Meier survival estimates for Parkinson’s disease for rugby players compared to the general population


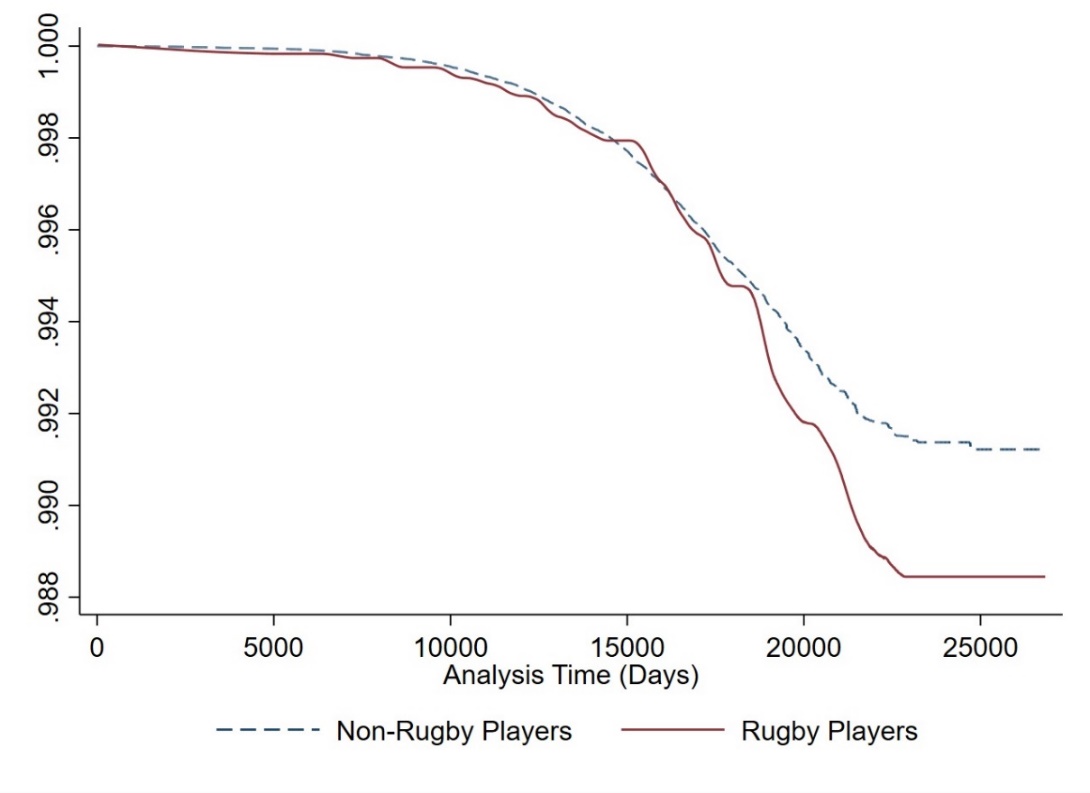


Note: As very few rugby players were diagnosed with MND the survival curve was smoothed in order to meet Stats NZ’s confidentiality requirements for statistical output release (Microdata output rule 4.8; StatsNZ 2020).

**Fig. S8** Kaplan-Meier survival estimates for Motor Neuron Disease for rugby players compared to the general population


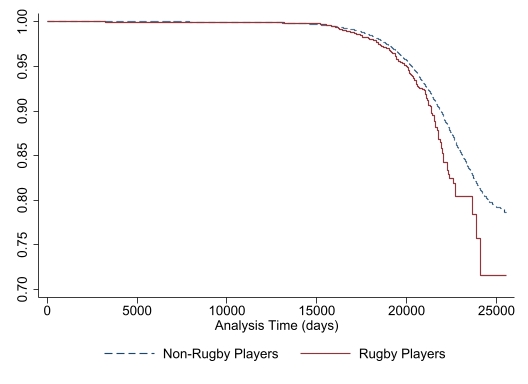


**Fig. S9** Kaplan-Meier survival estimates for mortality (main cause) from any neurodegenerative disease for rugby players compared to the general population


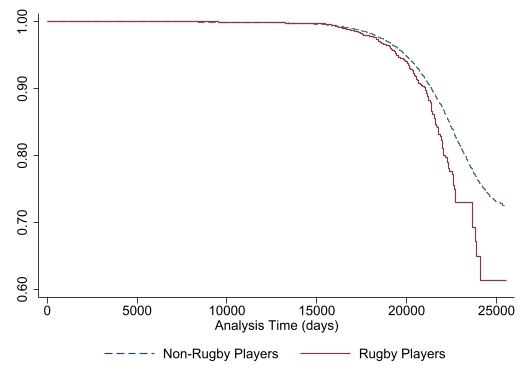


**Fig. S10** Kaplan-Meier survival estimates for mortality (including contributing cause) from any neurodegenerative disease for rugby players compared to the general population


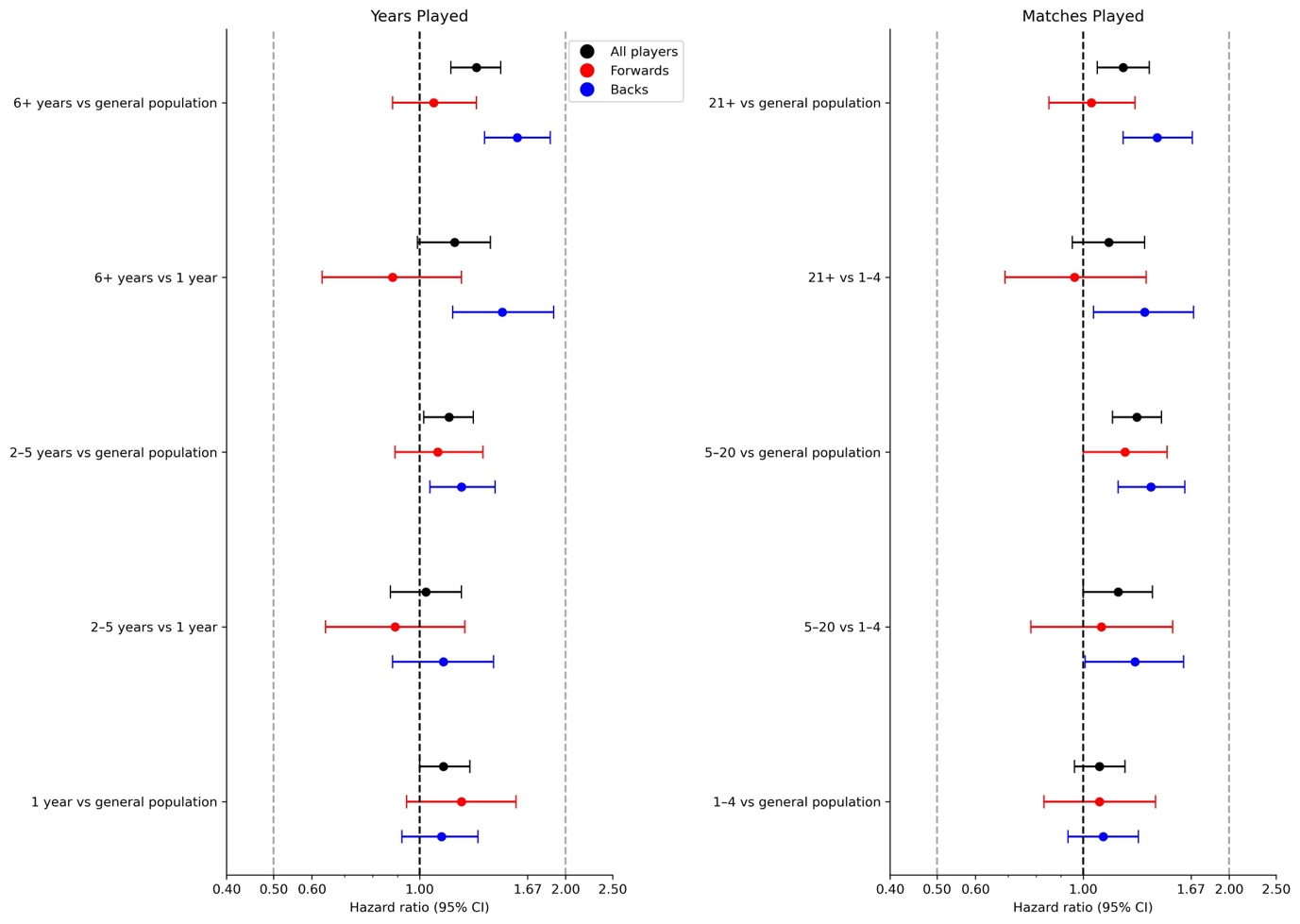


**Fig. S11** Hazards ratios for any neurodegenerative disease by exposure and playing position

**eReferences**

1. Te Whatu Ora. interRAI New Zealand. Annual report 2022/23 [Internet]. Wellington, New Zealand: Te Whatu Ora; 2023 p. 15. Available from: https://www.interrai.co.nz/assets/AR_interRAI_2023_FINAL_WEB-v2.pdf

2. Walesby KE, Exeter DJ, Gibb S, Wood PC, Starr JM, Russ TC. Prevalence and geographical variation of dementia in New Zealand from 2012 to 2015: Brief report utilising routinely collected data within the Integrated Data Infrastructure. Australas J Ageing. 2020;39:297–304.

3. Richmond-Rakerd LS, D’Souza S, Milne BJ, Caspi A, Moffitt TE. Longitudinal Associations of Mental Disorders With Dementia: 30-Year Analysis of 1.7 Million New Zealand Citizens. JAMA Psychiatry [Internet]. 2022 [cited 2022 Feb 17];79:333–40. Available from: https://jamanetwork.com/journals/jamapsychiatry/fullarticle/2789298

4. Pitcher TL, Myall DJ, Pearson JF, Lacey CJ, Dalrymple-Alford JC, Anderson TJ, et al. Parkinson’s disease across ethnicities: A nationwide study in New Zealand. Movement Disorders [Internet]. 2018 [cited 2023 Aug 25];33:1440–8. Available from: https://onlinelibrary.wiley.com/doi/abs/10.1002/mds.27389

5. Cao MC, Chancellor A, Charleston A, Dragunow M, Scotter EL. Motor neuron disease mortality rates in New Zealand 1992–2013. Amyotrophic Lateral Sclerosis and Frontotemporal Degeneration [Internet]. 2018 [cited 2024 Jun 17];19:285–93. Available from: https://doi.org/10.1080/21678421.2018.1432660
